# A nomogram prognostic model for diffuse large B‐cell lymphoma based on SUVmax and GNRI in elderly patients

**DOI:** 10.1002/jha2.794

**Published:** 2023-09-23

**Authors:** Maoqin Li, Haihao Lu, Jiaoyang Fan, Min Dai, Chang Su

**Affiliations:** ^1^ Department of Hematology The First Affiliated Hospital of Sun Yat‐sen University Guangzhou P. R. China

**Keywords:** diffuse large B‐cell lymphoma (DLBCL), elderly, geriatric nutritional risk index (GNRI), maximum standardized uptake value (SUVmax), nomogram

## Abstract

To establish a nomogram for elderly patients with diffuse large B‐cell lymphoma (DLBCL) based on nutritional and imaging features. The data of 221 elderly pretreatment DLBCL patients were retrospectively analyzed. All cases were randomly separated into the training group and validation group. A nomogram was built based on the results of multivariate analysis. A nomogram was established based on maximum standardized uptake value (SUVmax), geriatric nutritional risk index (GNRI), and lactate dehydrogenase. The concordance index (C‐index) of the nomogram was 0.772 for the training group and 0.729 for the validation group, and similar results were found in the area under the curve (AUC). The calibration curve showed favorable consistency between prediction and real survival. The decision curve analysis (DCA) also showed that the nomogram had favorable clinical effectiveness. The new risk‐stratification model divided patients into three groups with obvious survival. The C‐index and AUCs for the new model were greater than those of IPI and NCCN‐IPI. The DCA curve suggested that the new model had better clinical effectiveness than the IPI and NCCN‐IPI. The nomogram prognostic model based on SUVmax and GNRI performed superior to NCCN‐IPI and equal to IPI for risk stratification of elderly DLBCL patients.

## Graphical abstract

A nomogram prognostic model for elderly patients with diffuse large B‐cell lymphoma (DLBCL) was established based on maximum standardized uptake value (SUVmax), geriatric nutritional risk index (GNRI), and lactate dehydrogenase (LDH), as shown in Figure A. The new risk‐stratification model based on nomogram divided patients into three groups with obvious survival: the low‐risk group (0–119 points, 39.37%), intermediate‐risk group (132–193 points, 48.87%), and high‐risk group (219 points, 11.76%). The distribution of the three groups is shown in Figure B, and the Kaplan–Meier survival curve of the three groups is shown in Figure C.

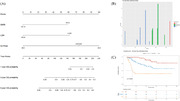

## 1 INTRODUCTION

Diffuse large B‐cell lymphoma (DLBCL) is a group of aggressive tumors with high heterogeneity in the clinical features, morphology, cell of origin, and molecular or cytogenetic features of DLBCL, and its prognosis is also remarkably variable [1]. According to the Surveillance, Epidemiology, and End Results (SEER) database, 54.6% of DLBCL patients were ≥65 years old at the time of diagnosis, and the median age was 66 years old [2], indicating that elderly patients were the main population. Compared with young people, the elderly has decreased organ function, more comorbidities, and worse nutritional and physical status. The 5‐year overall survival (OS) rate of elderly patients treated with the R‐CHOP regimen is 58% [3].

A multicenter clinical study showed that 14.7% of elderly inpatients had malnutrition and 35% had the risk of malnutrition [4]. Approximately 30–60% of cancer patients are malnourished [5]. It can be seen that the nutritional status of elderly cancer patients is poor, and clinicians should pay more attention to the impact of nutritional status on survival of patients. There are many tools used to evaluate the nutritional status of elderly patients, for example, the Mini Nutritional Assessment short‐form (MNA‐SF) [6]. But it is difficult to implement in clinical practice due to the complexity, subjectivity, and time‐consuming of such questionnaires. In addition, there are many objective and concise indicators have been proved to be associated with the prognosis of DLBCL patients, such as the serum albumin (ALB) [7], body mass index (BMI) [8], geriatric nutritional risk index (GNRI) [9], advanced lung cancer inflammation index (ALI) [10], and prognostic nutritional index (PNI) [11]. Therefore, this study intends to further verify the effects of GNRI, PNI, and ALI on the survival of elderly DLBCL patients. Imaging indexes derived from positron emission tomography/computed tomography (PET/CT) are not only used for staging and efficacy evaluation, but also for prognosis prediction in DLBCL [12]. The maximum standardized uptake value (SUVmax) can reflect the invasiveness of tumor.

The commonly used prognostic models for DLBCL include the international prognostic index (IPI) [13] and National Comprehensive Cancer Network international prognostic index (NCCN‐IPI) [14]. However, these two traditional models do not consider the impact of nutrition, imaging features, and cell of origin on the prognosis of patients. At present, there is a lack of prognostic model for elderly patients with DLBCL, building a more suitable prognostic model for elderly DLBCL has guiding significance for clinical diagnosis and treatment. Therefore, this study intends to analyze the impact of nutrition on the prognosis of elderly DLBCL through the GNRI, PNI, and ALI, and then establish a more suitable nomogram prognostic model and risk‐stratification model for elderly patients with DLBCL based on nutritional and imaging features.

## 2 MATERIALS AND METHODS

### 2.1 Subjects

We enrolled 221 pretreatment DLBCL patients who were ≥60 years old between January 2012 and April 2022 at the First Affiliated Hospital of Sun Yat‐Sen University. The diagnosis was mainly based on the corresponding version of the WHO classification of tumors of hematopoietic and lymphoid tissues. Most patients received first‐line R‐CHOP(like)‐based immunochemotherapy (rituximab plus cyclophosphamide, doxorubicin, vincristine, and prednisolone), and a small number of patients with HBV infection could not use rituximab and received CHOP(like) regimen. Exclusion criteria included (1) patients with less than two courses of chemotherapy; (2) patients with other malignancies (excluding those who were cured or had not relapsed within 5 years); and (3) primary central nervous system DLBCL, EB virus‐positive DLBCL, or transformed DLBCL.

Data collected from the electronic medical record system included sex, age, height, weight, comorbidities, Eastern Cooperative Oncology Group (ECOG) score, B symptoms, Ann Arbor stage classification, presence of bulky (≥7.5 cm) or extranodal tumor involvement, Ki‐67 expression, cell of origin (COO) via the Hans algorithm [15], neutrophil count, lymphocyte count, lactate dehydrogenase (LDH), albumin, and SUVmax. Inpatient medical records were reviewed, and follow‐ups were conducted over the telephone. OS is defined as the period from diagnosis to the date of death or last follow‐up.

### 2.2 Evaluation of nutrition status

The following equations were utilized to determine the patient nutritional status:

GNRI = 14.89 × albumin (g/dL) + 41.7 × (real weight/ideal weight),
ideal weight = 22 × [height (m)]^2^, when the weight ratio exceeds 1, the result is recorded as 1.
ALI = body mass index × albumin (g/dL)/(neutrophil count/lymphocyte count)
PNI = 10 × albumin (g/dL) + 5 × 10^−3^ × lymphocyte count (/mm^3^)

### 2.3 Statistical analysis

All cases were randomly separated into the training group (*n* = 155) and validation group (*n* = 66) according to the ratio of 7:3. The optimal cutoff value of prognostic parameters was obtained by receiver‐operating characteristic (ROC) curve with the maximum Youden index. The chi‐squared (χ^2^) test was used to compare the baseline characteristics between the two groups. Univariate analysis was performed in the training group to screen out the risk factors associated with OS (*P* < 0.05). Multivariate Cox stepwise regression analysis was used to obtain independent risk factors, and a nomogram was built based on the regression coefficients of independent risk factors. The model's performance was internally and externally verified by concordance index (C‐index), time‐dependent ROC curves, calibration curves, and decision curve analysis (DCA). The new risk‐stratification model based on nomogram was achieved by X‐tile software. Analyses were performed using R statistical software (version 4.2.1), and a *P* < 0.05 represented a statistically significant difference.

## 3 RESULTS

### 3.1 Clinical features and survival

Among 221 elderly DLBCL patients, 51.6% were male, the median age was 67 years (range, 60–88), and 23.1% were more than 75 years old. The mean BMI was 22.4 kg/m^2^ (range, 13.5–29.7), 43.9% had morbidities, and 16.3% were multimorbid (two or more comorbidities). Additionally, 61.5% of patients were already advanced‐stage at diagnosis, 78.7% had an ECOG PS score ≤1, 28.5% had B symptoms, 25.3% had bulky disease, and 38.0% had more than one extranodal site involved. Elevated LDH and non‐GCB occurred in 49.3% and 53.8% of patients, respectively. Up to 28.1% and 14.7% of patients were high‐risk based on IPI (≥4 scores) and NCCN‐IPI (≥6 scores), respectively. Further, 91.8% of patients received R‐CHOP(like) regimens, and 17.2% received relevant surgeries.

Calculating the cutoff values of each parameter by ROC curves, the cutoff for BMI was 20.6 kg/m^2^, SUVmax was 9.3, Ki‐67 expression was 55%, and GNRI, ALI, and PNI were 91.8, 28, and 44, respectively. In addition, 28.1% of patients had a BMI < 20.6 kg/m^2^, 57.5% had an SUVmax > 9.3, 28.9% missed SUVmax value, and 81.9% had a Ki‐67 expression > 55%. Patients with low GNRI, low ALI, and low PNI accounted for 30.3%, 47.1%, and 48.4%, respectively, as shown in Table 1.

**TABLE 1:** Clinical characteristics of elderly DLBCL patients and comparison between groups.

Characteristic *n*, (%)	Total *n* = 221	Training *n* = 155 (70)	Validation *n* = 66 (30)	*P*‐value
Gender				0.297
Female	107 (48.4)	71 (45.8)	36 (54.5)	
Male	114 (51.6)	84 (54.2)	30 (45.5)	
Age				1
≤75	170 (76.9)	119 (76.8)	51 (77.3)	
>75	51 (23.1)	36 (23.2)	15 (22.7)	
BMI				0.516
≥20.6	159 (71.9)	114 (73.5)	45 (68.2)	
<20.6	62 (28.1)	41 (26.5)	21 (31.8)	
Comorbidities				0.920
0–1	185 (83.7)	129 (83.2)	56 (84.8)	
≥2	36 (16.3)	26 (16.8)	10 (15.2)	
B symptoms				1
No	158 (71.5)	111 (71.6)	47 (71.2)	
Yes	63 (28.5)	44 (28.4)	19 (28.8)	
ECOG				0.868
0–1	174 (78.7)	123 (79.4)	51 (77.3)	
2–5	47 (21.3)	32 (20.6)	15 (22.8)	
Stage				0.347
Limited	85 (38.5)	56 (36.1)	29 (43.9)	
Advanced	136 (61.5)	99 (63.9)	37 (56.1)	
Bulky				0.679
NO	165 (74.7)	114 (73.5)	51 (77.3)	
Yes	56 (25.3)	41 (26.5)	15 (22.7)	
Extranodal site				0.900
≤1	137 (62.0)	97 (62.6)	40 (60.6)	
≥2	84 (38.0)	58 (37.4)	26 (39.4)	
SUVmax^a^				0.102
≤9.3	30 (13.6)	20 (12.9)	10 (15.2)	
>9.3	127 (57.5)	96 (61.9)	31 (47.0)	
Unknow	64 (28.9)	39 (25.2)	25 (37.9)	
SUV of bone marrow^a^				0.947
Normal	128 (77.6)	96 (77.4)	32 (78.0)	
Abnormal	23 (13.9)	17 (13.7)	6 (14.6)	
Reactive	14 (8.5)	11 (8.9)	3 (7.3)	
COO				0.991
GCB	102 (46.2)	71 (45.8)	31 (47.0)	
Non‐GCB	119 (53.8)	84 (54.2)	35 (53.0)	
Ki‐67				0.188
≤55	40 (18.1)	32 (20.6)	8 (12.1)	
>55	181 (81.9)	123 (79.4)	58 (87.9)	
LDH				0.546
≤ULN	112 (50.7)	76 (49.0)	36 (54.5)	
>ULN	109 (49.3)	79 (51.0)	30 (45.5)	
GNRI				1
≥91.8	140 (63.3)	98 (63.2)	42 (63.6)	
<91.8	81 (36.7)	57 (36.8)	24 (36.4)	
ALI				0.671
≥28	117 (52.9)	84 (54.2)	33 (50.0)	
<28	104 (47.1)	71 (45.8)	33 (50.0)	
PNI				0.454
≥44	114 (51.6)	83 (53.5)	31 (47.0)	
<44	107 (48.4)	72 (46.5)	35 (53.0)	
IPI^a^				0.771
Low	49 (27.5)	34 (27.0)	15 (28.8)	
Low‐intermediate	41 (23.0)	27 (21.4)	14 (26.9)	
Intermediate‐high	38 (21.3)	29 (23.0)	9 (17.3)	
High	50 (28.1)	36 (28.6)	14 (26.9)	
NCCN‐IPI^a^				0.923
Low‐intermediate	73 (41.2)	52 (41.6)	21 (40.4)	
Intermediate‐high	78 (44.1)	54 (43.2)	24 (46.2)	
High	26 (14.7)	19 (15.2)	7 (13.5)	
Chemotherapy^a^				0.975
R‐CHOP(like)	134 (91.8)	96 (92.3)	38 (90.5)	
CHOP(like)	12 (8.2)	8 (7.7)	4 (9.5)	
Dose				1
Standard	60 (41.1)	43 (41.3)	17 (40.5)	
Reduced	86 (58.9)	61 (58.7)	25 (59.5)	
Surgery				0.106
Yes	38 (17.2)	22 (14.2)	16 (24.2)	
No	183 (82.8)	133 (85.8)	50 (75.8)	

ALI, advanced lung cancer inflammation; BMI, body mass index; COO, cell of origin; ECOG, Eastern Cooperative Oncology Group; GNRI, geriatric nutritional risk index; IPI, international prognostic index; LDH, lactate dehydrogenase; NCCN‐IPI, National Comprehensive Cancer Network international prognostic index; PNI, prognostic nutritional index; SUVmax, maximum standardized uptake value; ULN, upper limit of normal value.

^a^ There were 64 patients missed SUVmax value, and those patients were analyzed as an SUVmax unknown group. Forty‐three patients missed IPI and NCCN‐IPI, and 75 patients missed chemotherapy regimens.

**FIGURE 1:**
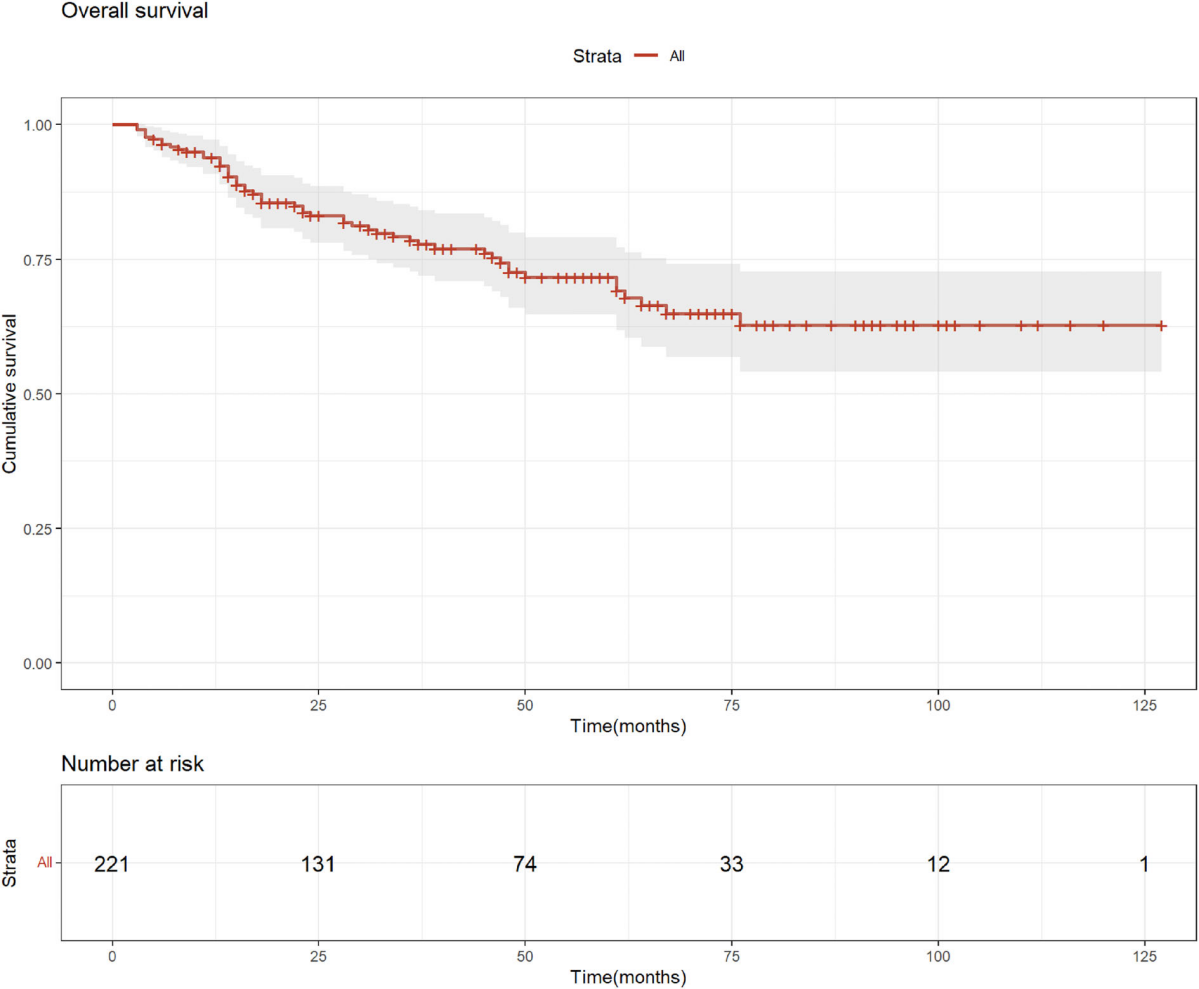
Kaplan–Meier survival curve for all cases.

The survival of elderly patients was optimistic, with a median follow‐up of 4 years. The 1‐year‐OS rate in all patients was 92.4% (95%CI: 88.9%–96.1%), the 3‐year‐OS rate was 78.5% (95% CI: 72.7%–84.7%), and the 5‐year OS rate was 69.1% (95% CI: 61.9%–77.2%), as shown in Figure 1.

### 3.2 Nomogram construction

**FIGURE 2:**
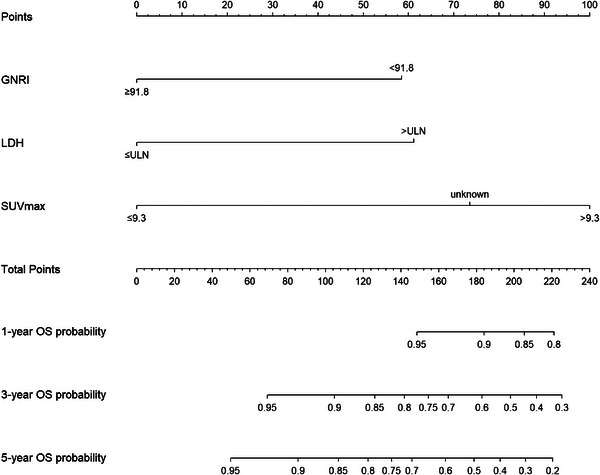
The nomogram prognostic model. GNRI, geriatric nutritional risk index; LDH, lactate dehydrogenase; SUVmax, maximum standardized uptake value.

Potential factors, including advanced stage, two or more extranodal sites, LDH > ULN, SUVmax > 9.3, GNRI < 91.8, and ALI < 28, were obtained by univariate analysis of the training group, as shown in Table 2. Given the GNRI and ALI are the same types of indicators for nutritional or immunological status, to avoid collinearity interference between variables, GNRI and ALI were assigned to two subgroups: group A included GNRI, stage, extranodal site, SUVmax, and LDH; group B included ALI and other four factors. Multivariate analysis showed that GNRI < 91.8, LDH > ULN, and SUVmax > 9.3 were significant variables affecting prognosis in group A, and only LDH > ULN in group B. Model A (0.772) was superior to model B (0.670, *P* < 0.001) in line with the C‐index. Finally, a nomogram was built for predicting 1‐, 3‐, and 5‐year‐OS upon three variables: GNRI < 91.8, LDH > ULN, and SUVmax > 9.3, as shown in Figure 2. The point of the three variables was then calculated based on coefficients in the multivariate analysis: SUVmax > 9.3 scored 100 points, SUVmax unknown scored 74 points, LDH > ULN scored 61 points, and GNRI < 91.8 scored 58 points.

**TABLE 2:** Cox regression analysis of training group.

Variables	Univariate		Multivariate	
	HR (95% CI)	*P*‐value	HR (95% CI)	*P*‐value
Gender				
F				
M	1.791 (0.920–3.487)	0.086		
Age				
≤75				
>75	1.725 (0.856–3.479)	0.127		
BMI				
≥20.6				
<20.6	1.669 (0.867–3.212)	0.125		
Comorbidities				
0–1				
≥2	2.089 (0.989–4.41)	0.054		
B symptoms				
No				
Yes	1.805 (0.953–3.42)	0.070		
ECOG				
0–1				
≥2	1.939 (0.980–3.837)	0.057		
Stage				
Limited				
Advanced	2.208 (1.047–4.657)	0.038	1.188 (0.506–2.786)	0.692
Bulky				
No				
Yes	1.278 (0.656–2.489)	0.47		
Extranodal site				
0–1				
>1	2.148 (1.143–4.036)	0.018	1.474 (0.723–3.005)	0.286
SUVmax^a^				
≤9.3				
>9.3	8.7 (1.184–63.96)	0.034	7.555 (1.025–55.676)	0.047
Unknown	6.0 (0.758–47.41)	0.090	4.424 (0.558–35.088)	0.159
SUV of marrow^a^				
Normal	1.317 (0.499–3.476)	0.579		
Abnormal Reactive	0.867 (0.259–2.898)	0.816		
COO				
GCB				
Non‐GCB	1.31 (0.695–2.471)	0.404		
Ki‐67				
≤55				
>55	1.243 (0.548–2.817)	0.602		
LDH				
≤ULN				
> ULN	3.452 (1.679–7.096)	<0.001	3.438 (1.656–7.140)	<0.001
GNRI				
≥91.8				
<91.8	2.694 (1.428–5.086)	0.002	3.259 (1.689‐6.288)	<0.001
ALI				
≥28				
<28	1.9 (1.003–3.599)	0.049		
PNI				
≥44				
<44	1.628 (0.866–3.06)	0.13		
Chemotherapy^a^				
R‐CHOP(like)				
CHOP(like)	1.708 (0.518–5.631)	0.379		
Dose				
Standard dose				
Reduced dose	0.546 (0.264–1.13)	0.103		
Surgery				
No	0.723 (0.257–2.039)	0.54		
Yes				

ALI, advanced lung cancer inflammation; BMI, body mass index; COO, cell of origin; ECOG, Eastern Cooperative Oncology Group; GNRI, geriatric nutritional risk index; LDH, lactate dehydrogenase; PNI, prognostic nutritional index; SUVmax, maximum standardized uptake value; ULN, upper limit of normal value.

^a^ There were 64 patients missed SUVmax value, and those patients were analyzed as an SUVmax unknown group. Forty‐three patients missed IPI and NCCN‐IPI, and 75 patients missed chemotherapy regimens.

### 3.3 Internal and external validation for the nomogram

**FIGURE 3:**
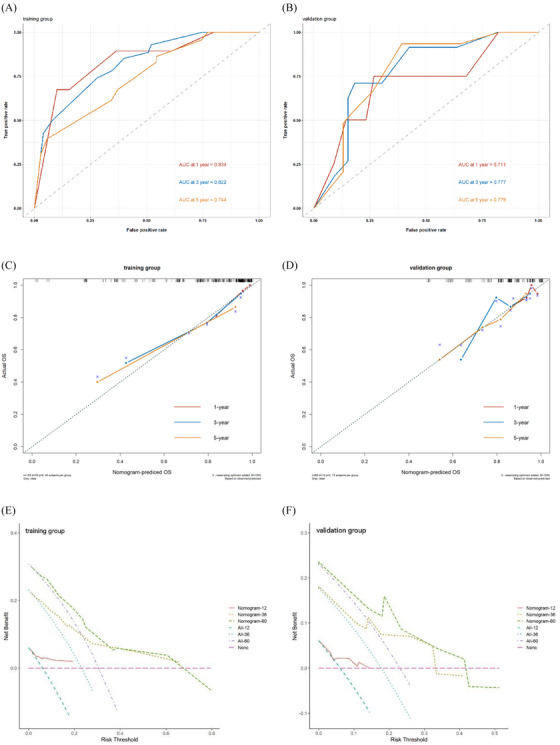
Internal and external validation of the nomogram prognostic model in elderly patients with DLBCL. (a, b) The time‐dependent receive‐operating characteristic (ROC) curves at 1‐, 3‐, and 5‐year‐OS in the training (a) and validation group (b). (c, d) The calibration curves for predicting 1‐, 3‐, and 5‐year‐OS in the training (c) and validation group (d); the actual OS is plotted on the *y*‐axis, and the model‐predicted probability of OS is plotted on the *x*‐axis. (e, f) The decision curve analysis (DCA) at 1‐, 3‐, and 5‐year‐OS in the training (e) and validation group (f); the abscissa represents the threshold probability, and the ordinate represents the net benefit. The DCA curves are above two straight dashed lines, indicating that the prediction model has a good net benefit.

C‐index, time‐dependent ROC curves, calibration curves, and DCA curves were used to validate the nomogram performance. The C‐index of the model was 0.772 (95% CI, 0.700–0.843) for the training group and 0.729 (95% CI, 0.590–0.868) for the validation group. Figure 3a,b demonstrates that the AUCs were 0.843, 0.822, and 0.744 at 1‐, 3‐, and 5‐year‐OS for the training group and 0.711, 0.777, and 0.779 for the validation group. Figure 3c,d illustrates that the calibration curves exhibit favorable uniformity between prediction and real survival in both groups. The DCA curves also showed that the nomogram model had favorable clinical net benefit in both groups, as shown in Figure 3e,f.

### 3.4 Comparison of the nomogram model with IPI and NCCN‐IPI

**FIGURE 4:**
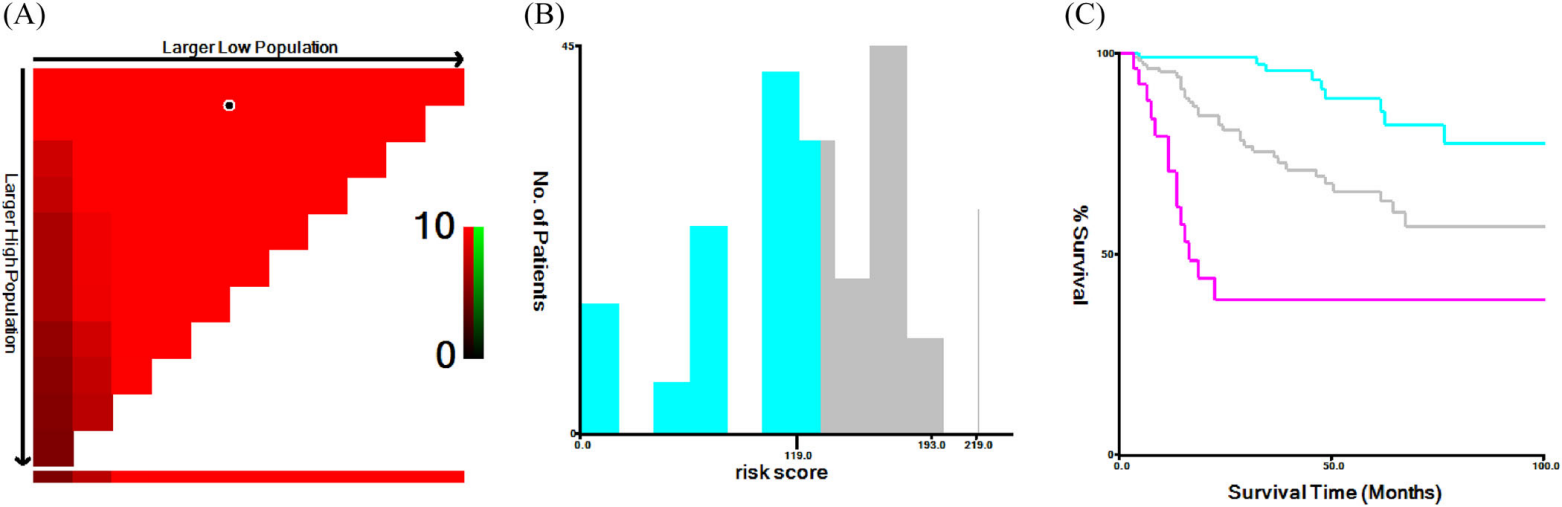
Three risk groups were identified by X‐tile based on the nomogram risk points for all patients. (a) Clicking on the triangle area can generate two optimal cutoff values: 119 and 193. (b) The distribution of the three groups divided by 119 and 193. (c) The Kaplan–Meier survival curves of the three groups.

Based on the nomogram risk points, cases were separated into three categories with obvious OS by X‐tile: the low‐risk group (points: 0–119, 39.37%), intermediate‐risk group (points: 132–193, 48.87%), and high‐risk group (points: 219, 11.76%; Figure 4). The 1‐year‐OS rate of the three groups was 98.9% (95%CI: 96.6%–100%), 94.3% (95%CI: 89.9%–98.8%), and 70.5% (95%CI: 54.2%–91.6%); the 3‐year‐OS rates were 95.6% (95%CI: 90.7%–100%), 74.2% (95%CI: 65.5%–84.1%), and < 38.5%; and the 5‐year‐OS rates were 85.6% (95%CI: 75.8%–96.7%), 63.4% (95%CI: 53%–75.9%), and < 38.5%, as shown in Figure 5a.

FIGURE 5Comparison of the new risk‐stratification model with IPI and NCCN‐IPI. (a–c) The Kaplan–Meier survival curves of the new risk‐stratification model (a), IPI (b) and NCCN‐IPI (c). (d–f) The time‐dependent receive‐operating characteristic (ROC) curves at 1‐ (d), 3‐ (e), and 5‐year‐OS (f) of the new risk‐stratification model, IPI, and NCCN‐IPI. (g–i) The decision curve analysis (DCA) at 1‐ (g), 3‐ (h), and 5‐year‐OS (i) of the new risk‐stratification model, IPI, and NCCN‐IPI. The curve of the new risk‐stratification model is above the curves of IPI and NCCN.

**Figure:**
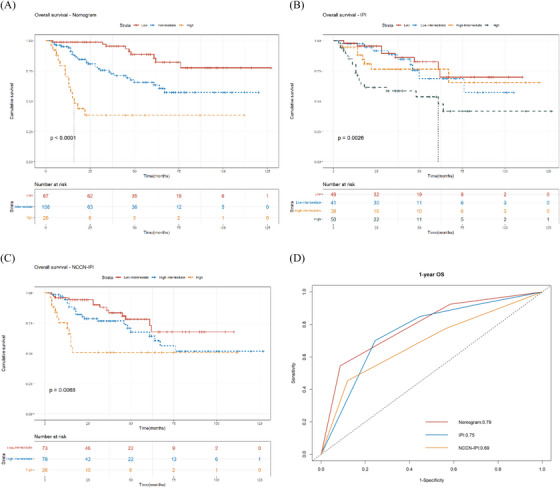

**Figure:**
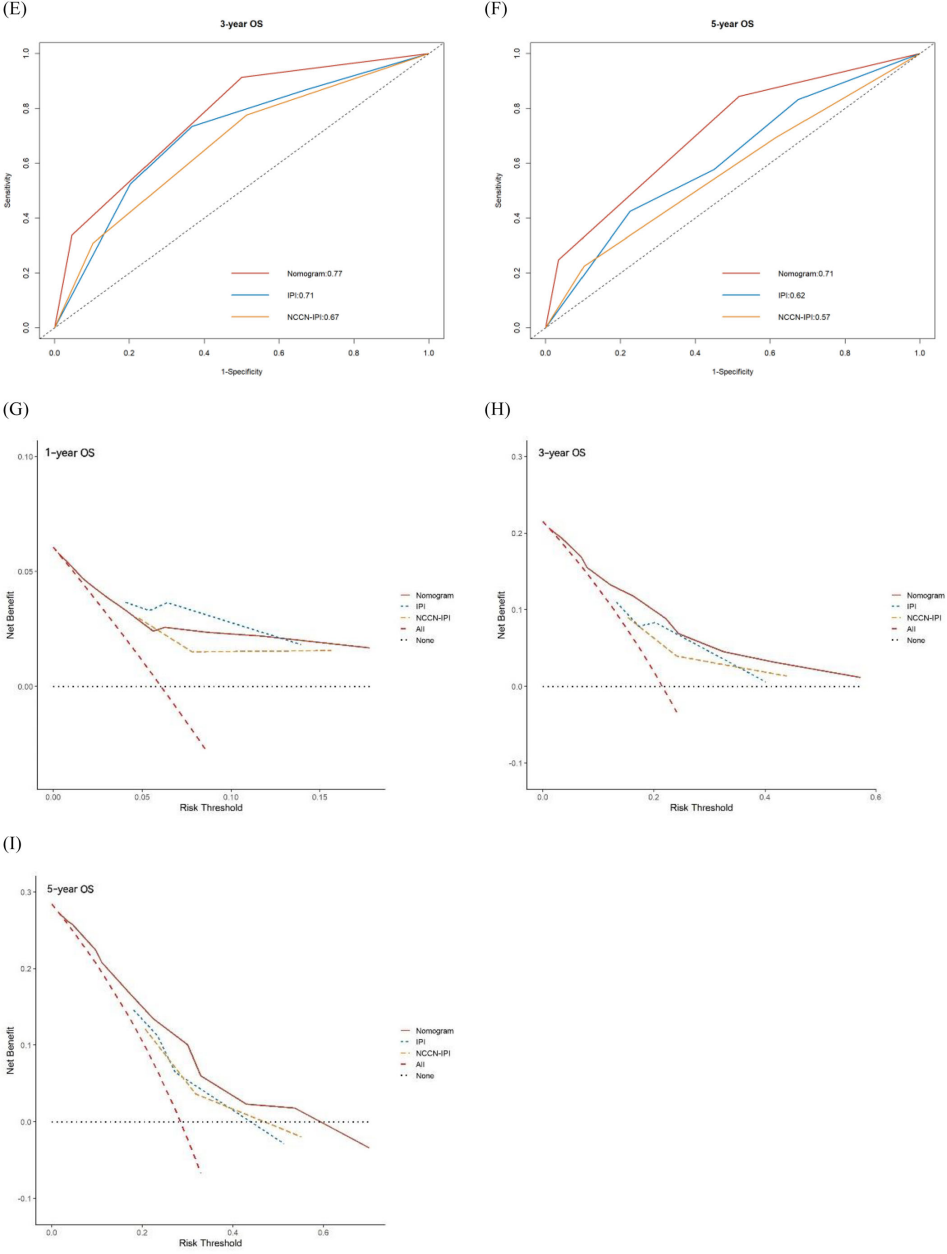

The Kaplan–Meier survival curves demonstrated that the new risk‐stratification model outperforms the other two models. Figure 5b,c demonstrates that each group of the two models has intersections or converging trends. The C‐index for the risk‐stratification model (0.729, 95%CI: 0.669–0.789) was greater than that of the IPI (0.679, 95%CI: 0.602–0.756, *P* = 0.091), and was significantly greater than that of the NCCN‐IPI (0.648, 95%CI: 0.562–0.734, *P* = 0.044). The predicted AUCs of the risk‐stratification model at 1‐, 3‐, and 5‐year ‐OS were also greater than that of the IPI (1 year‐OS: 0.79 vs. 0.75, *P* = 0.659; 3 year‐OS: 0.77 vs. 0.71, *P* = 0.100; 5 year‐OS: 0.71 vs. 0.62, *P* = 0.052), and were significantly greater than that of the NCCN‐IPI (1 year‐OS: 0.79 vs. 0.69, *P* = 0.160; 3 year‐OS: 0.77 vs. 0.67, *P* = 0.010; 5 year‐OS: 0.71 vs. 0.57, *P* = 0.004), as shown in Figure 5d–f. The DCA curves of the risk‐stratification model at 3‐ and 5‐year‐OS had better clinical effectiveness than the IPI and NCCN‐IPI, as shown in Figure 5g–i. The above results indicate that the nomogram model performed equal to IPI and superior to NCCN‐IPI for risk stratification of elderly DLBCL patients.

## 4 DISCUSSION

At present, the definition of elderly lymphoma has not been unified, and young and elderly patients are mostly distinguished by 60 years old [3, 16–18] or 65 years old [9, 19]. Elderly DLBCL were defined in this study as being greater than or equal to 60 years old. A nomogram was built to predict the prognosis of elderly DLBCL patients, which consisted of three variables from clinical practice, including GNRI, LDH, and SUVmax. The new risk‐stratification model based on the nomogram is convenient and objective with favorable discriminability, and with the performance equal to IPI and superior to NCCN‐IPI.

The GNRI, which contains albumin, weight, and height, was first proposed in 2005 to estimate the nutritional condition of aged patients [20]. The association of low GNRI with poorer prognosis in newly diagnosed elderly DLBCL patients has been reported in several studies [9, 16, 19], which is consistent with this study. Low GNRI is associated with poor clinical characteristics, such as aging, poor physical status, high LDH levels, and high disease stage [9]. The cutoff value (91.8) calculated by the ROC in this study is also numerically close to previous results, but lower than the domestic research report [16]. We also discussed other nutrition‐related indexes that may influence the prognosis of DLBCL patients. ALI, which first served as a mark for evaluating the prognosis of nonsmall‐cell lung cancer [21], is a significant unfavorable index for DLBCL [10, 22]. In this research, univariate analysis revealed that low ALI (<28) has an adverse prognostic effect in elderly patients, but it was not significant in multivariate analysis. The PNI, calculated by albumin and ALC, was proposed in 1980 to estimate the surgical risk for gastrointestinal patients [23]. Although numerous studies have explored its impact on survival for DLBCL, the significance of PNI remains disputed [11, 24–26]. Our results indicated that PNI with a cutoff value of 44 had no impact on predicting the survival of elderly DLBCL patients. Considering that the above differences are related to the different characteristics of study population, it can be further verified in larger studies.

PET/CT has been widely used in the evaluation of lymphoma, SUVmax, ΔSUVmax, MTV, TLG, and other imaging indicators can predict the prognosis of DLBCL [27]. High baseline SUVmax (>9) was an unfavorable prognostic factor affecting OS in DLBCL patients, but was not significant in multivariable analysis [28]. Another study showed that SUVmax was a significant risk variable affecting the survival of DLBCL patients with good guidance for prognostic prediction [29]. Conversely, a recent review found that most of the studies that considered the baseline SUVmax have no obvious predictive effect for OS in DLBCL. However, the forest plot result from that review found that SUVmax was statistically significant for evaluating survival [12]. Because some patients could not afford an expensive PET/CT scan, the sample size of previous PET/CT‐related studies is small. In this study, 157 elderly DLBCL patients had SUVmax values, and 64 patients with missing data were analyzed as an unknown group to ensure the integrity of the study. In this study, a high SUVmax level (>9.3) was a highly relevant predictor of poor survival in elderly DLBCL. However, the cutoff value of SUVmax varies among different studies and is disputed. In clinical practice, some basic hospitals have not yet popularized PET/CT equipment, and some patients could not afford the expensive inspection cost. This model serves as a warning for improving patients’ cognition of DLBCL prognosis.

In univariate analysis, except ECOG PS ≥2 (*P* = 0.057), elevated LDH, advanced stage, and two or more extranodal involvement sites were adverse indexes of OS (*P* < 0.05), but only elevated LDH was significant in the multivariate analysis, which may be owing to the small sample size in this study. It also indicated that LDH is the most significant of the five risk factors of IPI, which was consistent with a previous study. The study found that elevated LDH is the most frequently distributed and the highest prognostic impact among the five factors of IPI, and patients with elevated LDH have lower complete remission (CR) rate, event‐free survival (EFS), and OS regardless of IPI score [30].

There are several limitations to our model. First, the significance of the SUVmax unknown group included in this nomogram needs further verification. Second, the nomogram did not include molecular or genetics indicators. Third, this research was designed as a single‐center retrospective study with small sample size, and thus, our results need to be further verified in a larger multicenter study.

## 5 CONCLUSION

(1) The nomogram prognostic model based on SUVmax and GNRI has good discrimination, calibration, and clinical effectiveness, and can accurately evaluate the survival of elderly DLBCL patients. (2) The new risk‐stratification model based on the nomogram prognostic model is equal to IPI and superior to NCCN‐IPI for prognosis prediction, risk stratification, and clinical effectiveness of elderly patients with DLBCL.

## AUTHOR CONTRIBUTIONS

Chang Su designed the research study and revised this paper critically. Maoqin Li performed the research, analyzed the data, and wrote the paper. Haihao Lu collected the data and wrote the paper. Jiaoyang Fan collected and analyzed the data. Min Dai collected and analyzed the data.

## FUNDING INFORMATION

There is no funding for this study.

## CONFLICT OF INTEREST STATEMENT

All authors declare no conflict of interest.

## PATIENT CONSENT STATEMENT

This study has applied for an exemption from signing the informed consent of the subjects.

## PERMISSION TO REPRODUCE MATERIAL FROM OTHER SOURCES

The materials in this study are all original and do not need permission.

## CLINICAL TRIAL REGISTRATION (INCLUDING TRIAL NUMBER)

This study is not a clinical trial.

## Data Availability

The data that support the findings of this study are available from the corresponding author upon reasonable request.
